# Validation of Visual Estimation of Portion Size Consumed as a Method for Estimating Food Intake by Young Indian Children

**Published:** 2007-03

**Authors:** Pratibha Dhingra, Sunil Sazawal, Venugopal P. Menon, Usha Dhingra, Robert E. Black

**Affiliations:** ^1^ Center for Micronutrient Research, Annamalai University, Chidambaram, India; ^2^ Department of International Health, Johns Hopkins Bloomberg School of Public Health, 615 North Wolfe Street, Baltimore, MD 21205, USA

**Keywords:** Food intake, Caloric intake, Infant, Child, Visual estimation, Food record, Observational studies, India

## Abstract

In this observational study, estimation of food intake was evaluated using recording of portion size consumed, instead of post-weighing, as a method. In total, 930 feeding episodes were observed among 128 children aged 12–24 months in which actual intake was available by pre- and post-weighing. For each offering and feeding episode, portion size consumed was recorded by an independent nutritionist—as none, less than half, half or more, and all. Using the pre-weighed offering, available intake was estimated by multiplying portion sizes by the estimated weight. The estimated mean intake was 510.4 kilojoules compared to actual intake of 510.7 kilojoules by weighing. Similar results were found with nestum (52.0 vs 56.2 g), bread (3.8 vs 3.7 g), puffed rice (1.7 vs 1.9 g), banana (31.3 vs 24.4 g), and milk (41.6 vs 44.2 mL). Recording portion size consumed and estimating food intake from that provides a good alternative to the time-consuming and often culturally-unacceptable method of post-weighing food each time after a feeding episode.

## INTRODUCTION

Methods currently used for assessing the dietary practices of children are maternal 24-hour recall, food diaries, dietary histories, food-frequency recalls, semi-quantitative food-frequency recalls, and the techniques of estimating portion sizes of food intakes by weighing, visual estimation, or by applying standard portion sizes (1). All these methods have significant shortcomings (2, 3). Multiple studies have shown that parental recall of child's food intake excludes a large part of non-mealtime intake (4). The direct observation of food intake among young children is often considered only an appropriate method in natural or cafeteria settings (6–8), overcomes the potential limitations of maternal recall and influence of literacy levels of respondents, and is less likely to alter feeding behaviour of young children (9). The commonly-used and accepted method of pre- and post-weighing of foods during observation is cumbersome and, in many cultures, unacceptable; in addition, it may actually modify the intake by drawing attention of caretaker (10). Direct observation using visual estimation is a non-intrusive method of estimating dietary intake and may provide a better alternative (11–14). In direct observation, trained personnel observe food-consumption behaviour and visually estimate food intake, and the accuracy of estimation varies by type and quantity of food (6). Observation can be labour-intensive; it is, therefore, usually done either for only one meal or for one day's meal (14, 15). The validity of direct observation using visual estimates for measuring food intake has been evaluated in few studies in developed-country settings (7, 8, 16). Data on validation of these techniques in developing-country field-settings are lacking. In a study, where food intake from a test meal was estimated by pre- and post-weighing of all foods the child consumed, we evaluated the direct visual estimation of portion size consumed as a method for estimating food intake.

## MATERIALS AND METHODS

The study was conducted in a periurban population of New Delhi, India during September 1993-March 1994. In total, 130 children aged 12–24 months were enrolled after obtaining verbal informed consents from the parents of the children. The human research review committees of the All India Institute of Medical Sciences and the Johns Hopkins Bloomberg School of Public Health approved the study procedures.

Trained dieticians observed each child at home for six hours. A standardized pre-weighed test meal, consisting of six items (nestum mixed in lactogen [rice-based weaning food from Nestlé India Ltd.]–200 g [cooked], milk–200 mL [boiled], puffed rice–20 g, banana–75 g, and bread–50 g), was given to the mother on the morning before the start of observation. Foods offered to the child during the feeding episodes were recorded by weighing every offering. As part of the investigation, leftover and spillage were also recorded using the standard methods. Actual intake was estimated from pre- and post-weighing of foods by subtracting food recovered or wasted during feeding for each test meal item from the food offered to the child by the mother.

For the direct observation exercise, the feeding episode was observed by an independent observer, who was not involved in pre- and post-weighing of foods and was blinded to actual weighing data. This observer recorded, for six hours, interactions of mother and child, and a question regarding the portion size of every food consumed in each feeding episode was added to the appetite-assessment data-collection tool (17). For every feeding episode, a separate sheet was filled, and, within each sheet, there was a row for each food offered. The portion size consumed by the child for each food offered was coded as 1 for all or most food consumed, 2 for half or more consumed, 3 for less than half consumed, and 4 for none consumed. During the estimation/analysis for each food in a feeding episode, using data from pre-weighed offering by the mother, food intake for each food was estimated by multiplying offering by portion consumed—[Estimated intake=Quantity offered by mother × portion size consumed by the child (coded 1=1, 2=0.75, 3=0.5, and 4=0).

The SPSS software (version 12.0) was used for statistical analyses. Pearson's correlation coefficients and paired *t*-test were estimated to examine the associations between the estimates of food intake derived from the visual estimation method and the actual recorded food intake by post-weighing.

## RESULTS

The sample characteristics of the study population, including age, nutritional status, gender, breastfeeding status, and energy intake before the start of observation, and illness on the day of observation are presented in Table 1. In total, 930 feeding episodes in 128 children were observed. The estimated intake based on the visually-observed method and recorded caloric intake and intake of individual test meal food items using post-weighing were similar (Table 2).

**Table 1.** Sample characteristics of study population

Characteristics	n=128
Age (months)*	17.3±4.4
Age-group (months) (%)	
≤15	41.4
16–19	25.8
≥20	32.8
Nutritional status (%)	
Stunted	40.6
Wasted	7.0
Wasted+stunted	8.6
Male (%)	52.3
Breastfed (%)	92.2
Intake before observation (energy, KJ)*	475±378

*Mean±SD;

KJ=Kilojoules;

SD=Standard deviation

**Table 2.** Association of estimated vs recorded food intake of children (n=128)

Food	No. of feeding observations	Mother offered/g (mean±SD)	Estimated intake/g (mean±SD)	Actual intake/g (mean±SD)	Variation in estimation		Correlation
					(±5%)	(±10%)	
Nestum	195	168.2±61.1	52.0±49.3	56.2±62.2	14.9	20.8	0.83
Bread	50	9.56±15.6	3.8±8.3	3.7±8.3	26.3	50.0	0.93
Puffed rice	83	4.51±7.1	1.7±3.2	1.9±3.6	26.7	26.7	0.61
Banana	119	58.7±43.0	31.3±36.8	24.4±28.0	9.9	15.5	0.81
Milk	99	65.3±82.1	41.6±66.2	44.2±68.4	41.2	42.6	0.93
Overall*	930		510.4±316.4	510.7±321.4			0.88

*Total food intake estimated from test meal and other foods consumed

The difference between the two provides data for other foods consumed

## DISCUSSION

The results of the study suggest that food intake estimated by visual observation and estimation is a useful and valid method for assessing dietary intake of children. This method yields very similar estimates of food intake as that of recorded food intake by post-weighing and overcomes the limitations and problems associated with post-weighing of foods during observation periods, which, in many cultures, is an unacceptable practice and may possibly alter behaviour. These findings are similar to findings of earlier studies conducted in institutional settings (8, 9, 13, 14, 18) and support the validity of the visual estimation method for measuring portion sizes. Most earlier studies have been carried out among school children, either in natural or cafeteria settings, to measure food intake by estimating the plate waste in school lunch and their eating behaviour but data relating to estimating the food intake in preschool children are not available. We evaluated the technique with a set of five most commonly-consumed foods that were offered for the test meal. The fact that a lower proportion of offered food for puffed rice and bread was consumed suggests that children were either not used to or did not like these two foods; however, for estimation purposes, they behaved similar to the other three foods.

Although these findings need to be confirmed for other foods, there is no reason to believe that results with other foods not evaluated in the present study will be different. If these findings are confirmed with other foods, it offers a very useful method of assessing food intake for young preschool children in settings that allow for direct observation food intake but requires minimum disruption of the eating environment. To our knowledge, this is the first study carried out among young children in a field setting to evaluate the validity of visual estimation of portion size consumed and weighing only the food offered during the observation periods. The results are very encouraging and need confirmation in other settings with other foods.

In conclusion, a direct observation method with weighed offering only and estimating intake with a simple coding for portion size consumed, training for which can be imparted easily to field or health workers, is feasible and is an accurate method for estimating total food intake of young children.

## ACKNOWLEDGEMENTS

This work was supported by grants from the World Health Organization, Thrasher Research Fund, and United States Agency for International Development Cooperative Agreement No. NIH.R29HD34724. The authors are grateful to the parents of the participating children for their time and cooperation.
